# Prevalence of lung structure abnormalities in patients with acromegaly and their relationship with gas exchange: cross-sectional analytical study with a control group: Prevalência de alterações na estrutura pulmonar em pacientes com acromegalia e sua relação com a troca gasosa: estudo transversal analítico com grupo controle

**DOI:** 10.1590/1516-3180.2013.7640012

**Published:** 2014-09-19

**Authors:** Marcelo Palmeira Rodrigues, Luciana Ansaneli Naves, Carlos Alberto Viegas, Cesar Augusto Melo-Silva, Wagner Diniz de Paula, Márcia Teixeira Cabral, Renata Rodrigues Araújo, Luiz Augusto Casulari

**Affiliations:** I MD, MSc, PhD. Adjunct Professor of Pneumology, School of Medicine, Universidade de Brasília (UnB), Brasília, Brazil; II MD, MSc, PhD. Adjunct Professor of Endocrinology, School of Medicine, Universidade de Brasília (UnB), Brasília, Brazil; III MSc, PhD. Physiotherapist, Department of Pneumology, Hospital Universitário de Brasília (HUB), Brasília, Brazil; IV MD. Physician, Department of Radiology, Hospital Universitário de Brasília (HUB), Brasília, Brazil; V MD. Physician, Department of Pneumology, Hospital Universitário de Brasília (HUB), Brasília, Brazil; VI MD, MSc, PhD. Physician, Department of Endocrinology, Hospital Universitário de Brasília (HUB), Universidade de Brasília (UnB), Brasília, Brazil

**Keywords:** Acromegaly, Growth hormone, Anoxia, Lung, Tomography, X-ray computed, Acromegalia, Hormônio do crescimento, Anóxia, Pulmão, Tomografia computadorizada por raios X

## Abstract

**CONTEXT AND OBJECTIVE:**

Different functional respiratory alterations have been described in acromegaly, but their relationship with pulmonary tissue abnormalities is unknown. The objective of this study was to observe possible changes in lung structure and explain their relationship with gas exchange abnormalities.

**DESIGN AND SETTING:**

Cross-sectional analytical study with a control group, conducted at a university hospital.

**METHODS:**

The study included 36 patients with acromegaly and 24 controls who were all assessed through high-resolution computed tomography of the thorax (CT). Arterial blood gas, effort oximetry and serum growth hormone (GH) and insulin-like growth factor I (IGF-1) were also assessed in the patients with acromegaly.

**RESULTS:**

The abnormalities found in the CT scan were not statistically different between the acromegaly and control groups: mild cylindrical bronchiectasis (P = 0.59), linear opacity (P = 0.29), nodular opacity (P = 0.28), increased attenuation (frosted glass; P = 0.48) and decreased attenuation (emphysema; P = 0.32). Radiographic abnormalities were not associated with serum GH and IGF-1. Hypoxemia was present in seven patients; however, in six of them, the hypoxemia could be explained by underlying clinical conditions other than acromegaly: chronic obstructive pulmonary disease in two, obesity in two, bronchial infection in one and asthma in one.

**CONCLUSION:**

No changes in lung structure were detected through thorax tomography in comparison with the control subjects. The functional respiratory alterations found were largely explained by alternative diagnoses or had subclinical manifestations, without any plausible relationship with lung structural factors.

## Abstract (pt)

**CONTEXTO E OBJETIVO:**

Diferentes alterações funcionais respiratórias são descritas na acromegalia. Sua relação com anormalidades do tecido pulmonar é desconhecida. O objetivo foi observar possíveis alterações da estrutura pulmonar e explicar sua relação com anormalidades da troca gasosa.

**TIPO DE ESTUDO E LOCAL:**

Estudo transversal, analítico, com grupo de controle, realizado em um hospital universitário.

**MÉTODOS:**

Incluíram-se 36 pacientes com acromegalia e 24 controles que foram avaliados com tomografia computadorizada de alta resolução de tórax (TC); os acromegálicos também foram avaliados com gasometria arterial, oximetria de esforço e dosagens de hormônio de crescimento (GH) e fator de crescimento semelhante à insulina (IGF-1).

**RESULTADOS:**

As alterações encontradas na TC não foram estatisticamente diferentes entre os grupos acromegálico e de controle: bronquiectasia cilíndrica leve (P = 0,59), opacidades lineares (P = 0,29), opacidades nodulares (P = 0,28), aumento da atenuação (vidro fosco) (P = 0,48) e redução da atenuação (enfisema; P = 0,32). As alterações radiológicas não se relacionaram com as dosagens de GH e IGF-1. Hipoxemia estava presente em sete pacientes; contudo, em seis deles a hipoxemia poderia ser explicada por condição clínica subjacente diversa da acromegalia: doença pulmonar obstrutiva crônica em dois, obesidade em dois, infecção brônquica em um e asma em um.

**CONCLUSÕES:**

Não foram observadas alterações da estrutura pulmonar por tomografia de tórax, quando comparadas ao grupo de controle; as alterações funcionais respiratórias encontradas são explicáveis em grande parte por diagnósticos alternativos, ou se manifestam de forma subclínica, não apresentando relação plausível com o aspecto da estrutura pulmonar.

## INTRODUCTION

Patients with acromegaly have higher respiratory mortality rates when stratified according to gender and age.^1^ It has been reported that patients with acromegaly die prematurely and that mortality due to respiratory diseases is three times more frequent than would be expected in the entire population.^1^ In that retrospective study, the type of respiratory disease leading to death was not described. A relative mortality risk due to respiratory diseases of 1.85, in comparison with that of the general population, has been described in the literature. This risk increases to 2.32 when the growth hormone (GH) level remains high after treatment, i.e. between 2.5 ng/ml and 9.9 ng/ml.^2^

Many respiratory functional abnormalities have been described in patients with acromegaly. Increased lung volume was initially described in the 1970s.^3^–^5^ There is evidence that GH levels are associated with lung volume. Patients with hypopituitarism and GH deficiency have lung volume reductions in comparison with acromegaly patients.^6^ There is some controversy regarding the reason for the increase in lung volume. It has been suggested that it could be due to the increased number of alveoli.^7^ However, it has been observed that the increase in lung compliance found in patients with acromegaly was minimized or even disappeared after hormonal control, thus indicating that the reason for the greater lung volume was the increased alveolar size and not their number.^8^

Static lung volumes depend on the elastic properties of the respiratory system and muscle strength. Some patients with acromegaly show decreases in respiratory muscle strength, although the magnitude of this change has not been significantly correlated with the increased in lung volume, or with measurements of partial pressure of arterial carbon dioxide (PaCO_2_) and oxygen (PaO_2_).^9^

Increased airflow resistance in patients with acromegaly, with reduced flows at low lung volumes, has also been observed, which suggests that the small airways are involved,^5^ as well as clearly obstructive lung disease in nonsmoking patients with acromegaly.^10^

Studying gas exchange is particularly important in this context. On the one hand, it is the result of the sum of variables that determines ventilation, diffusion and the ventilation-perfusion balance, among others. On the other hand, major gas exchange alterations such as severe hypoxemia are directly involved in morbidity and mortality rates.^11^

Varying levels of hypoxemia with increased alveolar-arterial oxygen gradients have been described in patients with acromegaly, thereby suggesting the existence of disturbance of the ventilation/perfusion relationship.^12^ This finding has not been observed by other authors.^8^,^9^ However, it should be noted that the samples of that study were small and there was no investigation of stress-induced hypoxemia.

To our knowledge, despite the functional alterations observed, no patients with acromegaly have been evaluated for possible alterations in the lung structure using high-resolution computed tomography of the thorax. Only a few observations resulting from simple chest X-rays with normal lung parenchyma have been reported.^4^,^12^

## OBJECTIVE

The aim of this study was to investigate whether patients with acromegaly show lung structure alterations, in comparison with control subjects, and whether these possible alterations are related to gas exchange abnormalities.

## METHODS

### Patients

Patients with acromegaly who were treated at the Neuroendocrine Unit of Hospital Universitário de Brasília underwent examinations as part of a cross-sectional analytical study with a control group. The diagnosis of acromegaly was established based on clinical characteristics and the following biochemical findings:^13^ lack of growth hormone (GH) suppression (less than 1 μg/l) during the standard oral glucose tolerance test using 75 g of anhydrous glucose; and levels of insulin-like growth factor I (IGF-I) that were considered high for the age and gender. The sample included patients with both controlled and uncontrolled acromegaly, as shown by a normal IGF-I or glucose tolerance test. Patients presenting uncontrolled hypopituitarism were excluded from the study.

A control group was included to compare the data obtained via high-resolution computed tomography (CT) of the thorax, with data from other patients who underwent this examination at Hospital Universitário de Brasília within the period of this study. The exclusion criteria for the control group were clinical evidence of respiratory tract disease and acromegaly. This was a convenience sample and it was not matched for sex and age. However, the samples were matched for age and sex by coincidence. Information on age, sex and smoking habit was collected from all patients.

GH levels (Immulite 2000, chemiluminescent enzyme immunometric methodology, Diagnostic Products Corporation, Los Angeles, CA, USA) and IGF-I levels (radioimmunoassay after extraction) were used in comparisons involving radiological abnormalities and oxygen level.

### Functional respiratory examination

Arterial blood gas analysis was performed by collecting blood from the radial artery, with examination via gasometry for less than three minutes (AVL Compact 3, USA). The reference value for the partial pressure of arterial oxygen (PaO_2_)^14^ was corrected for the altitude of Brasilia, such that the average atmospheric pressure at the place of examination was 680 mmHg. The lower limit was obtained by subtracting the predicted value, which was 1.96 times the standard error of the estimate.

In conducting the exercise oximetry, a magnetic-type bicycle ergometer (DJ168 Venus, Belo Horizonte, Brazil) was used. Increasing effort up to the maximum time of six minutes was required. The exercise started without weights and continued until it reached a level close to 60 W. The test was immediately suspended if a decrease of 4% in peripheral oxygen saturation (SpO_2_) in hemoglobin was observed. A pulse oximeter was used (Healthdyne 950, USA) for SpO_2_ readings.

### Radiological examination

The radiological study was carried out using a CT scanner (Light Speed QX/I, GE Medical Systems, Milwaukee, WI, USA). High spatial resolution sections were made for observations of the lung parenchymal structure. The technique followed sequential acquisition at maximum inspiratory apnea, with collimation of 2 × 0.625 mm, increments of 10 mm and a reconstruction algorithm of high spatial frequency, without intravenous administration of exogenous contrast agent.

The findings were consensually described by two radiology experts who did not have any previous knowledge of the clinical or functional data of each patient, and followed standardized nomenclature.^14^ The findings were grouped into bronchiectasis, linear opacities, nodular opacities, increased attenuation, decreased attenuation, atheromatous aorta, coronary alterations and vertebral degenerative abnormalities. Small high-density nodular opacities (calcified granuloma) were excluded from the analysis.

### Statistical analysis

Student's t test was used for statistical analysis of independent samples and, when pertinent, for comparison of variables between the two groups of patients, i.e. with and without acromegaly. Evaluation of the normal distribution of each variable was done using the Shapiro-Wilk test. When the variable under assessment did not meet the criteria for normal distribution of values, the Mann-Whitney test was used. With regard to nominal data, the chi-square test was used for proportions. The variables were expressed as means ± standard deviations when the data showed normal distribution. Otherwise, the variables were expressed as medians and interquartile ranges. The findings that were considered statistically significant were those in which the associated probability in two-tailed tests was P < 0.05. The statistical software used was the SPSS software for Windows, version 13.0.

### Ethics

The study design was approved by the Research Ethics Committee of the School of Medicine of the University of Brasília. All participants (acromegaly and control groups) gave their written informed consent in order to participate in the study.

## RESULTS

The sample initially included 19 male and 17 female patients with acromegaly. The mean age was 49 ± 12.5 years (range: 24-67 years). The mean time elapsed since the diagnosis of acromegaly was 6.9 ± 5.2 years (range: 7 months to 25.1 years).

Except for three individuals (8.3%) who were included in the study prior to treatment for acromegaly, all the patients had previously undergone the following therapeutic approaches: surgery, radiotherapy and octreotide administration (n = 13; 36.1%); surgery and octreotide administration (n = 8; 22.2%); primary treatment with octreotide (n = 7; 19.4%); surgery only (n = 4; 11.1%); and surgery and radiotherapy (n = 1; 2.8%).

The control group included 24 patients, of whom 19 were volunteers in another protocol whose objective was to evaluate coronary alterations through CT. The other five patients had neoplasia: adenocarcinoma of the pancreas, colon, Hodgkin's lymphoma, gestational trophoblastic disease and basal cell carcinoma on the face. They underwent CT scans as part of a metastasis screening process.

**Table 1** shows potential confounders. There was no statistically significant difference between the groups with regard to age, gender or smoking habit. Smoking habit was assessed as a proportion of the patients or as the number of pack-years of smoking.

**Table 1:** Distribution of the acromegaly and control groups according to age, sex and smoking

Variables	Acromegaly (n = 36)	Control (n = 24)	P
Age (years)	49.5 ± 12	55.0 ± 17	0.15
Male subjects	53%	58%	0.67
Proportion of smokers	42%	48%	0.64
Pack-years of smoking	0 (0–12)	0 (0–20)	0.68*

* Mann-Whitney test (values expressed as medians and interquartile ranges).

CT of the pulmonary parenchyma showed several abnormalities in both groups, as observed in **Table 2**. In the group with increased attenuation, only ground-glass opacity was observed, whereas in the group with decreased attenuation, only emphysema-type lesions were seen. No nodular opacity had a diameter greater than 1 cm. The entire set of lesions observed was of small extent. If it had been possible to arrange each set of lesions in contiguity for a given patient, the area taken would have been less than one bronchopulmonary segment, except for emphysema, which was diffuse and extensive. When compared with the proportion of parenchymal lesions found in the control group, no statistically significant differences were found.

**Table 2:** Proportion of radiographic abnormalities in the acromegaly and control groups obtained by means of high-resolution computed tomography of the thorax

Radiological abnormality	Acromegaly (n = 36)	Control (n = 24)	P
Mild cylindrical bronchiectasis	22.2%	16.7%	0.59
Linear opacity	44.4%	58.3%	0.29
Nodular opacity	36.1%	50.0%	0.28
Increased attenuation (frosted glass)	13.9%	20.8%	0.48
Decreased attenuation (emphysema)	8.3%	16.7%	0.32
Coronary atheromatous disease	11.1%	50.0%	< 0.01
Vertebral degenerative alterations	83.3%	41.7%	< 0.01

With regard to non-pulmonary alterations seen on thorax tomography, a significant difference in the proportion of atheromatous alterations was observed. These were greater in the control group, and vertebral degenerative alterations were more pronounced in patients with acromegaly (**Table 2**).

Of the 36 patients initially included in the study, 3 refused to undergo arterial puncture for blood gas analysis. Of the 33 patients who underwent arterial blood gas analysis, 7 (21%) had PaO_2_ levels below the lower limit of normality, but none had levels of respiratory failure (PaO_2_ < 60 mmHg). Among the 7 patients with hypoxemia, 1 had bronchial asthma with symptoms on the examination day, 2 had chronic obstructive pulmonary disease, 2 were obese, 1 had bronchial infection and 1 had no clinically apparent respiratory disease in the assessment of symptoms or lung structure from the CT scan. In absolute terms, these patients showed PaO_2_ levels from 0.5 to 4.8 mmHg, i.e. below the lower limit.

**Figure 1:**
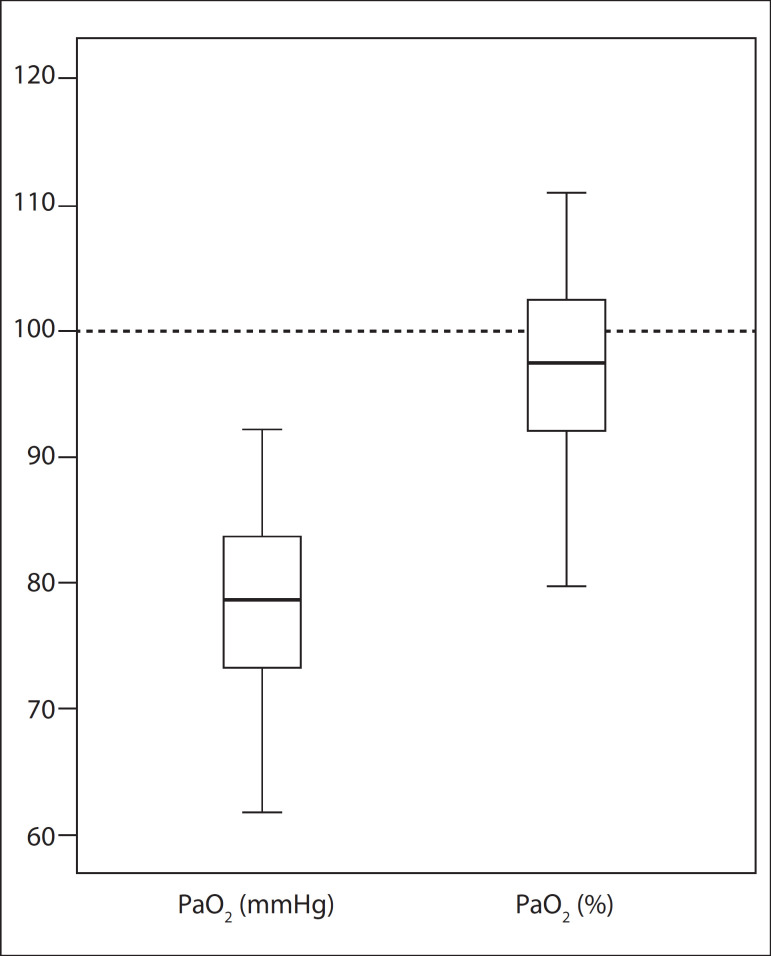
Distribution of absolute and relatively low PaO_2_ values, in mmHg. This was not confirmed when the values were corrected for the altitude of Brasília (PaO_2_ as % of predicted values).

**Figure 1** shows the distribution of absolute PaO_2_ values in mmHg, and they are relatively low. However, this was not confirmed when the values were corrected for the altitude of Brasília (PaO_2_ as % of predicted values).

Four patients did not undergo exercise oximetry: 1 refused to do it, 1 was excluded due to knee arthralgia; 1 was excluded due to severe sequelae from pelvic trauma; and 1 was excluded due to the presence of coronary disease. None of the 32 patients who did undergo exercise oximetry showed decreased SPO_2_ during exercise.

As shown in **Table 3**, the comparisons between PaO_2_, GH and IGF-I values as a function of the presence or absence of parenchymal lung abnormalities in each group of tomographic alterations were not statistically significant.

**Table 3:** PaO_2_, growth hormone (GH) and insulin-like growth factor I (IGF-I) values based on the presence or absence of radiological pulmonary abnormalities, obtained by means of high-resolution thorax computed tomography

Radiological abnormality		PaO_2_ (%)	P*	GH	P^†^	IGF-I	P^†^
Bronchiectasis	yes (n = 8)	97 ± 11	0.76	2.65 (0.94–5.2)	0.67	404 (155–604)	0.74
	no (n = 28)	96 ± 8		2.35 (0.8–4.45)		347 (184–536)	
Linear opacity	yes (n = 16)	95 ± 8	0.45	1.9 (0.4 -4.2)	0.34	372 (165–536)	0.92
	no (n = 20)	97 ± 9		2.52 (1- 4.7)		317 (214–587)	
Nodular opacity	yes (n = 7)	93 ± 4	0.26	3 (0.54–4.3)	0.70	322 (219–557)	0.95
	no (n = 29)	97 ± 9		2.2 (1–4.65)		372 (169–556)	
Increased attenuation	yes (n = 5)	97 ± 5	0.95	1 (0.2–3.37)	0.12	132 (85–446)	0.12
	no (n = 31)	96 ± 9		2.54 (1.1–4.5)		373 (224–557)	
Decreased attenuation	yes (n = 3)	104 ± 10	0.09	2.1 (2–14)	0.47	510 (360–565)	0.49
	no (n = 33)	96 ± 8		2.5 (0.9–4.3)		323 (176–538)	
Any alteration	yes (n = 25)	96 ± 9	0.58	2 (0.76–4.19)	0.17	322 (169–534)	0.53
	no (n = 11)	98 ± 8		3.1 (1.48–4.8)		408 (299–592)	

* Student t test;

† Mann-Whitney test.

## DISCUSSION

It has been well established that respiratory alterations in patients with acromegaly may be involved in higher morbidity and mortality rates associated with this disease.^1^,^15^ Acromegaly patients develop various respiratory alterations as a result of anatomical changes involving skull and facial bones and soft tissues, cartilage, respiratory mucosa, lung volume, rib cage geometry and respiratory muscle activity.^15^

In previous studies on the acromegaly patients who were the subject of the present study,^16^,^17^ it was observed that hypoxia during sleep (defined as more than five episodes of desaturation per hour) occurred frequently. It affected 41% of the enrolled patients. It was demonstrated that craniofacial abnormalities, obesity and GH and IGF-1 alterations have similar effects on the magnitude of the development of nocturnal hypoxemia in patients with acromegaly.^16^ Moreover, in evaluating clinical data as predictors of this condition, it was found that neck circumference greater than 44 cm was the main factor involved, in comparison with other commonly implicated factors such as body mass index (BMI), daytime sleepiness and snoring.^17^

In this study, the parenchymal lung abnormalities seen on high-resolution thorax tomography showed no statistically significant difference between the patients with acromegaly and the control group. Therefore, no pulmonary findings from this study can be associated with acromegaly. It is likely that factors common to both groups could be responsible, such as smoking. For instance, among other abnormalities related to smoking, emphysema is observed in both groups. In addition, it has been shown that the severity of sleep apnea syndrome in acromegaly patients is related to smoking and lung disease.^18^

Nevertheless, the association of pulmonary lesions with deaths among acromegaly patients is unclear.^15^ Out of 256 cases reviewed, chronic bronchitis and emphysema were found in five patients and bronchiectasis in three other patients.^19^ In another study on 1,362 acromegaly patients, only 11 of them died due to smoking, chronic bronchitis, emphysema and chronic obstructive pulmonary disease.^2^ These results are consistent with those observed in the present study, i.e. no increased frequency of these alterations was observed in either the acromegaly or the control patients. Corroborating this observation of a lack of relationship between acromegaly and lung structural alterations, there were also no significant differences in GH and IGF-I levels (**Table 3**), based on the presence or absence of radiological abnormalities in any of the lesion groups studied.

Two additional extrapulmonary observations were noted on high-resolution thorax tomography scans. The first was the much lower frequency of aortic and coronary atheromatous disease in the group with acromegaly, compared with that of the control group. In another study, no difference in the frequency of atheromatous plaques was found between acromegaly and control patients, although greater thickening of the intima and media layers of the carotid arteries was observed among those with active or inactive disease.^20^ It is probable that the results observed in the present study reflect significant selection bias in the control group, since the sample mainly included patients who had already been selected for investigation of vascular alterations.

The second observation was the much higher prevalence of vertebral degenerative alterations in the acromegaly patients, in comparison with the control group. Indeed, arthropathy is an important morbidity factor in these patients, affecting the axial skeleton about 52%,^21^ which is in agreement with the data observed.

In this study, the PaO_2_ levels were below the lower reference limit in seven patients (21%), of whom six had clinical conditions other than acromegaly to justify such values. Only one patient had no apparent reason for hypoxemia. It might be that this was a discrepant isolated case with an unusually low value, which is not uncommon in interpreting pulmonary function tests,^22^ and this may also have been associated with acromegaly or another unapparent condition.

The low hypoxemia magnitude found allows us to question its clinical relevance, particularly because of the hypothetical association between hypoxemia and respiratory morbidity and mortality in patients with acromegaly. In this study, no patient showed respiratory failure or decreased SpO_2_ during exercise and there was no relationship between hypoxemia and any radiological alterations in the lung structure.

Other authors have studied the behavior of PaO_2_ in acro-megaly.^8^,^9^,^12^ One study^8^ showed only the normal average value for the entire group. In another two studies, all of the individual values were described.^9^,^12^ Hypoxemia was present in only one of the ten acromegaly patients studied,^9^ when the same normality criteria used in the present study were applied.^23^ The other study included eleven acromegaly patients who were asymptomatic and nonsmokers, and who had normal thorax X-rays.^12^ Perfusion lung scans showed abnormal results in four out of five patients. Ten patients for whom PaO_2_ data were available showed hypoxemia prevalence of 80%. However, the authors did not define a lower normal limit criterion. Four of the patients were obese; one presented BMI of 39.3 kg/m^2^. When the normality criteria used in that study^23^ were applied to these patients, the hypoxia prevalence dropped to 70%.^12^ If PaO_2_ were to be corrected for BMI,^24^ the hypoxemia prevalence would be 50%, which is still a very high rate. It is possible that the patients studied by Luboshitzky et al.^12^ showed some pulmonary vascular involvement whose nature has not yet been correlated with the presence of acromegaly.

Hypoxemia has intra or extrapulmonary causes. The intra-pulmonary causes are diffusion and ventilation-perfusion disturbances. An association of hypoxemia with pulmonary alterations due to acromegaly in a cross-sectional study should be viewed with extreme caution, since ventilation-perfusion mismatches are present in a myriad of conditions. Therefore, it is necessary to rule out the coexistence of other diseases, particularly non-pulmonary diseases that cause hypoxia. Obesity, for example, can cause hypoxia through a zone of low ventilation-perfusion ratio formed in the lower regions due to changes in compliance of the thoracic cage.^25^

When functional respiratory alterations are subtle, a cross-sectional clinical investigation based on reference values makes it harder to distinguish whether alterations are present. Creating specific cutoff values to define functional alterations in acromegaly cases would first require definition of which abnormalities are clearly associated with this condition.

Longitudinal study designs are particularly interesting in this regard, since these make it possible to assess the impact of treatment on disease progression and simplify the issue of control, which may be due to the individual himself. However, it is very diﬃcult to define a homogeneous cohort with regard to the length of time of disease progression because the insidious nature of the disease leads to highly variable times between disease onset and diagnosis.

Although there is higher morbidity and mortality relating to lung diseases in patients with acromegaly, in comparison with healthy individuals,^1^ it is not clear which respiratory changes can contribute to this situation.^17^,^18^ The results reported in the present paper may contribute towards understanding the pulmonary function abnormalities of patients with acromegaly and may provide data for future research on this topic.

## CONCLUSION

In conclusion, no changes in lung structure were observed on thoracic tomography, in comparison with control subjects. The abnormalities in respiratory function found were largely explained by alternative diagnoses or had subclinical manifestations, without showing any plausible relationship with the abnormality of the lung structure.
